# The effects of community pharmacy public health interventions on population health and health inequalities: a systematic review of reviews protocol

**DOI:** 10.1186/s13643-017-0573-9

**Published:** 2017-08-30

**Authors:** Frances Hillier-Brown, Clare Bambra, Katie Thomson, Mirza Balaj, Nick Walton, Adam Todd

**Affiliations:** 1 Institute of Health and Society, Newcastle University, Richardson Road, Newcastle upon Tyne, NE2 4AX UK; 2 Department of Sociology and Political Science, Norwegian University of Science and Technology, Dragvoll, Building 9, Level 5, 7491 Trondheim, Norway; 3 School of Pharmacy, Faculty of Medical Sciences, Newcastle University, Queen Victoria Road, Newcastle upon Tyne, NE1 7RU UK

**Keywords:** Community pharmacy, Prevention, Health promotion, Public health, Health and health inequalities, Systematic review

## Abstract

**Background:**

Community pharmacies have great potential to deliver services aimed at promoting health and preventing disease, and are embedded within communities. In the light of a rapid increase in community pharmacy-delivered public health services and an accompanying increase in the evidence base, this systematic review of reviews will synthesise systematic reviews of public health community pharmacy interventions and assess their effects on public health and health inequalities.

**Methods/design:**

Systematic review methodology will be used to identify all systematic reviews that describe the health and health equity effects of community pharmacy public health interventions. Twenty databases will be searched using a pre-determined search strategy to evaluate community pharmacy-delivered public health interventions. Findings from the included reviews will be pooled, and a narrative synthesis executed to identify overarching patterns and results.

**Discussion:**

Findings will support future decision-making around how community pharmacy public health services can be used alongside other strategies to promote health, prevent disease and reduce health inequalities.

**Systematic review registration:**

PROSPERO registration number: CRD42017056264.

**Electronic supplementary material:**

The online version of this article (10.1186/s13643-017-0573-9) contains supplementary material, which is available to authorized users.

## Background

In recent years, community pharmacies have emerged as strategically important settings that have great potential to deliver services aimed at promoting health and preventing disease. Indeed, community pharmacies have, globally, been identified as an easily accessible and cost-effective platform for delivering healthcare services [1]. For example, in the UK, 89% of the population can reach a community pharmacy within 20 min and, crucially, in areas of high deprivation, that value increases to approximately 100% of the population—an observation known as the *positive pharmacy care law* [2]. In view of the wide accessibility to healthcare (a well-established social determinant of health), community pharmacies are ideally placed to offer public health services to all communities, including the most socio-economically deprived ones.

Considering this potential, the role of the community pharmacist has undergone rapid expansion [3]. Indeed, in addition to supplying medication, many community pharmacies now offer an abundance of patient-focussed public health services. Smoking cessation services were one of the earliest examples of such a service [4], but others, such as improving general lifestyle behaviours, increasing uptake of screening and giving sexual health advice, have since followed. Accompanying this development, the evidence base surrounding the extended role of the community pharmacist in public health has also expanded—with many groups producing systematic reviews examining the effectiveness of such interventions.

Despite this progress, however, and in view of the expanding literature base, the effects of community pharmacy interventions on population health—and perhaps more significantly, health inequalities—is still not clear. While there are many reviews, which focus on particular public health areas (e.g. [5] and [6]), these have not been brought together to assess the overall effects of the variety of community pharmacy interventions underway in the public health arena or how they impact on inequalities in health. It is, therefore, timely that we undertake a systematic review of reviews of community pharmacy services in order to support policy-makers and commissioners in their future decision-making around how community pharmacy public health services can be used alongside other strategies to promote health, prevent disease and reduce health inequalities.

## Methods

A systematic review of reviews will be conducted. This methodology is an established and effective way of bringing together and summarising a broad evidence base [7] and have been used for a number of public health topics [8–11]. Our review was designed using the Preferred Reporting Items for Systematic Review and Meta-Analyses (PRISMA) Guidelines [7]. A PRISMA-P checklist is available as an Additional file 1 to this protocol. This protocol is registered with PROSPERO (CRD42017056264).

### Research question

What are the effects of community pharmacy-delivered public health interventions on health and health inequalities?

### Study design

Systematic review methodology will be used to locate, appraise and synthesise published systematic review level evidence on the effects of community pharmacy-delivered interventions on health and inequalities in health [8–11].

### Inclusion criteria

Following standard evidence synthesis approaches [12], the inclusion criteria for the review are determined a priori in terms of PICOS (population, intervention, comparison, outcome and setting [13]).

- **Population**: Children and adults (all ages) in any country. The population is kept purposively broad to allow the widest range of literature to be identified.
- **Intervention**: Public health interventions delivered in community pharmacy settings. The inclusion criteria are purposely broad to allow for a range of different public health interventions to be located. For the purposes of the review, a community pharmacy was defined as a pharmacy set in the community, which is accessible to all and not based in a hospital, clinic or online [5].
- **Comparison**: We will include systematic reviews that include studies with and without controls, including randomised and nonrandomised controlled trials, randomised and nonrandomised cluster trials, prospective and retrospective cohort studies (with and/or without control groups), prospective repeat cross-sectional studies (with and/or without control groups) and interrupted time series (with and/or without control groups). Acceptable controls include randomised or matched designs. Reviews assessing qualitative studies will be excluded.
- **Outcomes**: Health and health inequality outcomes. Reviews that do not assess effectiveness of community pharmacy-delivered public health interventions will be excluded. Primary outcome measures will be conceptualised using the framework proposed by Hardeman and colleagues [14] and include determinants of behaviour (e.g. self-efficacy, perceived control), behavioural outcomes (e.g. smoking cessation, improved physical activity), physiology and biochemical outcomes (e.g. blood pressure, plasma cholesterol) and health outcomes (e.g. incidence rates of cardiovascular disease). Secondary outcomes relate to health inequalities in terms of PROGRESS-Plus factors: place of residence, race/ethnicity, occupation, gender, religion, education, socio-economic status (defined as individual income, wealth, education, employment or occupational status, benefit receipt; as well as area-level socio-economic indicators), social capital, age, disability and sexual orientation [15]. When available, cost effectiveness data will also be collected.
- **Setting**: Only systematic reviews will be included in the analysis. Included publications will need to meet the two mandatory criteria of Database of Abstracts of Reviews of Effects (DARE): (i) that there is a defined review question (with definition of at least two of, the participants, interventions, outcomes or study designs) and (ii) that the search strategy included at least one named database, in conjunction with either reference checking, hand-searching, citation searching or contact with authors in the field. When two reviews are identified with the same research aims (e.g. to assess the effectiveness of community pharmacy smoking cessation services), only the most recent review will be synthesised as part of this study.

### Search strategy

Twenty databases will be searched from inception until January 2017 (host sites given in parentheses): Medline (Ovid), Embase (Ovid), Cumulative Index to Nursing and Allied Health Literature (CINAHL; EBSCOhost), PsycINFO (EBSCOhost), Social Science Citation Index (Web of Science), Applied Social Sciences Index and Abstracts (ASSIA; ProQuest), International Bibliography of the Social Sciences (IBSS; ProQuest), Sociological Abstracts (ProQuest), Social Services Abstracts (ProQuest), Prospero (Centre for Reviews and Dissemination, University of York), Campbell Collaboration Library of Systematic Reviews (The Campbell Library), Cochrane Library (includes Cochrane Database of Systematic Reviews, Cochrane Central Register of Controlled Trials, Cochrane Methodology Register, Database of Abstracts of Reviews of Effects, Health Technology Assessment Database, NHS Economic Evaluation Database; Wiley), Database of Promoting Health Effectiveness Reviews (DoPHER; EPPI-Centre), Social Care Online (SCIE) and Health Systems Evidence. The search strategies have been developed from two previous systematic and umbrella review search strategies [2, 11] and an example search strategy is shown for Medline in Additional file 2. To ensure the sensitivity of the searches, five articles [5, 6, 16–18] known to the authors that met the inclusion criteria were identified. The search strategy was piloted in each of the databases and all of the five articles were retrieved. In addition, the bibliographies and reference lists of all included articles will be searched for further relevant publications. Searches will be limited to peer-reviewed publications only. Reviews will not be excluded based on language or publication date. If reviews are missing key information (e.g. information relating to study design or intervention details), they will be excluded from further analysis; however, attempts will be made to contact authors to obtain any relevant information that is missing.

### Screening, data extraction and quality appraisal

The initial screening of titles and abstracts will be conducted in duplicate by one reviewer (FHB) using EndNote reference management software (Thomson Reuters, Philadelphia, USA). A random 10% sample of titles and abstracts will be double screened by a second reviewer (NW) and inter-rater reliability will be assessed using the kappa statistic. Any discrepancies will be resolved through discussion between the two reviewers, and, if consensus is not reached, with the project lead (AT). Full text copies of potentially relevant articles will then be examined for inclusion by one reviewer (FHB), and checked by a second reviewer (AT).

Data will be extracted by one reviewer (NW or MB) and checked by a second (FHB), using standard data extraction forms based on previous reviews [8, 11]. Any discrepancies will be resolved through discussion between the two reviewers and the project lead (AT) if necessary. The following data will be extracted: the intervention type reviewed; the study population in the review (and in the included studies); who delivered the intervention; study design of studies included in the review (e.g. RCTs, controlled prospective cohort, repeat cross sections); any time/language/country/population restrictions in the review; the number of relevant studies in review (total); number of databases searched (total); whether grey literature was searched or citation follow up conducted; the method of synthesis (meta-analysis or narrative); any details on implementation of interventions contained within the review; and the main findings both in terms of health and socio-economic inequalities in health.

The methodological quality of each review will be determined using the Assessment of Multiple Systematic Reviews (AMSTAR) [19], which will be included as part of the data extraction form. The AMSTAR asks questions on: ‘a priori’ design, duplicate study selection and data extraction, literature search details, status of publications included, included and excluded study reference lists, characteristics of included studies, scientific quality of included studies, methods of combining findings, assessment of publication bias and conflict of interest. This is now the most frequently recommended tool for use within an systematic reviews of reviews, due to its ease of use and external validation [10, 13]. The use of AMSTAR also allows us to determine if the risk of bias was assessed in the included reviews. Further to this, we will use the revised version of AMSTAR (R-AMSTAR) to quantify the systematic review quality in terms of low, medium and high [20].

### Synthesis

A narrative synthesis will be conducted following the Economic and Social Research Council (ESRC) guidance, as described by Popay and colleagues [21]. We shall examine similarities and differences between findings of the included reviews, using effect sizes where meta-analysis has been conducted, and exploring patterns in narrative syntheses. Findings in terms of population level health effects will be described, as well as health inequality implications from any PROGRESS-Plus subgroup analyses reported. We will also assess each systematic review to determine if there are any overlapping studies. If overlapping studies are found, when undertaking the synthesis, we will consider systematic reviews that: (1) provide the most complete description, (2) are the most recent, (3) contain the most evidence and, (4) use the method that is the most vigorous. Dealing with overlapping studies has been acknowledged as a challenge when undertaking systematic reviews of reviews, [22] but our proposed approach to deal with such issues has been described previously [23]. Review quality, strength of evidence, and risk of bias will be considered in the interpretation of the findings, which is often lacking in systematic reviews of reviews [24].

## Discussion

This umbrella review will provide evidence how community pharmacy public health interventions affect population health and health inequalities. In addition, we will also establish and highlight any gaps in the systematic review evidence base around community pharmacy public health interventions. We anticipate the findings of this review will be used to by policy-makers and commissioners to inform future public health services; the review will also be used by academics to direct future research toward any evidence gaps that we may highlight.

## Additional files

Additional file 1: PRISMA-P 2015 Checklist. (DOC 82 kb)

Additional file 2: (MEDLINE, Ovid) Search strategy. (DOCX 21 kb)

## Abbreviations

AMSTAR: Assessment of multiple systematic reviews
DARE: Database of abstracts of reviews of effects
PICOS: Criteria for inclusion and exclusion of studies
PRISMA-P: Preferred reporting items of systematic reviews and meta-analyses protocol
PROGRESS-Plus: Acronym to identify population and individual characteristics across which health inequities may exist
PROSPERO: International prospective register of systematic reviews
R-AMSTAR: Revised version of AMSTAR
RCT: Randomised controlled trial
