# Supplementary material for: The effects of community pharmacy public health interventions on population health and health inequalities: a systematic review of reviews protocol
Source: Syst Rev. 2017 Aug 30;6:176. doi: 10.1186/s13643-017-0573-9 (PMC5577831; doi:10.1186/s13643-017-0573-9)
Supplement: Supplementary file 2 — (MEDLINE, Ovid) Search strategy. (DOCX 21 kb) [file 13643_2017_573_MOESM2_ESM.docx]

**Additional file 2:** (MEDLINE, Ovid) Search strategy

| **#** | **search term** |
| --- | --- |
| 1 | search$.tw. |
| 2 | (systematic adj2 (review$ or overview$)).mp. or Systematic Review/ |
| 3 | (meta analy$ or metaanaly$).mp. or Meta Analysis/ |
| 4 | (umbrella adj2 review).tw. |
| 5 | "review of reviews".tw. |
| 6 | 1 or 2 or 3 or 4 or 5 |
| 7 | exp Community Pharmacy Services/ |
| 8 | Pharmacies/ |
| 9 | exp Pharmacists/ |
| 10 | exp Pharmacists' Aides/ |
| 11 | Pharmacy/ |
| 12 | chemist.tw. |
| 13 | (communit$ adj7 pharmac$).tw. |
| 14 | (office$ adj7 pharmacy$).tw. |
| 15 | ((pharmacy or pharmacist? or pharmacies) adj3 (community or counsel$ or advice or care)).tw. |
| 16 | (pharmacist? adj3 (front line or 'one to one' or face to face)).tw. |
| 17 | (pharmacist? or pharmacy or pharmacies).tw. |
| 18 | ((pharmacist? or pharmacy) adj3 (aide or aides or assistant? or staff)).tw. |
| 19 | (Pharmacist? adj2 (care or delivered)).tw. |
| 20 | (pharmacist? adj3 (counsel$ or (patient? adj2 education$) or led or intervention? or public health or diagnos$)).tw. |
| 21 | or/7-20 |
| 22 | 6 and 21 |
| 23 | animals/ |
| 24 | humans/ |
| 25 | 23 not (23 and 24) |
| 26 | 22 not 25 |
